# Iron Overload and Platelet Function Defects: Possible Correlation

**DOI:** 10.1177/2324709616675645

**Published:** 2016-10-26

**Authors:** Abdulkader A. Dahi, Ehab Hanafy, Mohammed Al Pakra

**Affiliations:** 1 King Salman Armed Forces Hospital, Tabuk, Kingdom of Saudi Arabia

**Keywords:** iron overload, acquired platelet function defect, hemochromatosis, blood transfusion

## Abstract

Acquired platelet function defect might be a consequence of iron overload. Even though there are various complications of iron overload, only few reports have indicated some correlations with platelets dysfunction. We report a child with Diamond-Blackfan anemia who has significant complications from iron overload due to chronic blood transfusion, and one of these complications is acquired platelet function defect that manifests with frequent episodes of epistaxis. Therefore, we emphasize the necessity for further studies to confirm direct correlation between iron overload as a causative agent and platelets dysfunction. And we recommend screening for platelets function in patients receiving chronic blood transfusion aiming at possible prevention of any life-threatening bleeding.

## Introduction

Iron overload constitutes a major problem in patients receiving regular blood transfusion. Patients with β-thalassemia, sickle cell anemia, and congenital and refractory anemias on chronic transfusion programs accumulate iron in various body organs. Untreated iron overload will eventually lead to damage of the liver, endocrine organs, and most seriously the heart.^1^ Acquired platelet function defect might be one of the complications of iron overload. This could occur indirectly through the effect of iron load on the liver and other organs or might occur due to effect of iron load on platelet function directly.

To date, certain causes such as medications, medical illnesses, and hematologic diseases are associated with acquired platelet function defects. However, little is known about the direct effect of iron overload on platelet function.

We report a child with Diamond-Blackfan anemia on regular blood transfusion with iron overload that is associated with acquired platelet function defect manifesting with repeated episodes of epistaxis.

## Case

We report the case of an 11-year-old boy, who had full term, spontaneous vaginal delivery, with intra-uterine growth retardation. In his first day of life, the child was found severely pale, hypoactive with poor suckling, and so he was admitted at the neonatal intensive care unit to rule out sepsis. He had lab works done indicating hemoglobin 6.2 gm/dL, white blood cells 11.2 × 10^3^/µL, platelets 327 × 10^3^/µL, mean corpuscular volume 97 fL, reticulocyte count 0.1%, and red blood cells 2.3 × 10^6^/µL. Glucose 6-phosphate dehydrogenase was normal, hemoglobin electrophoresis was normal, TORCH screening was negative, and other lab works were unremarkable.

The child received supportive treatment, transfused with blood, and was put under follow-up for 2 months after which bone marrow aspirate was done, which supported the diagnosis of Diamond-Blackfan anemia.

He was started on steroids with no improvement of anemia, so he was planned for bone marrow transplantation; however, the parents refused this management despite several counseling sessions.

The child’s requirements for blood started to increase, so he was scheduled for a monthly blood transfusion program with iron chelation therapy in form of subcutaneous deferoxamine, which was replaced later with oral deferasirox.

At the age of 9, he developed diabetes mellitus, which is controlled with insulin. Shortly after that, he presented several times in the emergency room complaining of nose bleed. Initial lab works showed completely normal coagulation profile (prothrombin time, partial thromboplastin time, thrombin time, and fibrinogen), normal platelets count, and normal Von Willbrand assay. Further investigations were done including platelet function analyzer (PFA 100), which suggested platelet function disorder; collagen/EPI was 300+ seconds (normal is 92-180), and collagen/ADP was 256 seconds (normal is 67-127). At that point in time, serum ferritin level had exceeded 2000 ng/mL despite maximum dose of deferasirox and monthly intravenous (IV) deferoxamine.

The child received platelets transfusion with no response as an attempt to control the frequent episodes of epistaxis. Therefore, we started him on recombinant factor VII as prophylaxis every 10 days, which showed a satisfactory control of bleeding over the last 2 years.

**Figure 1.**
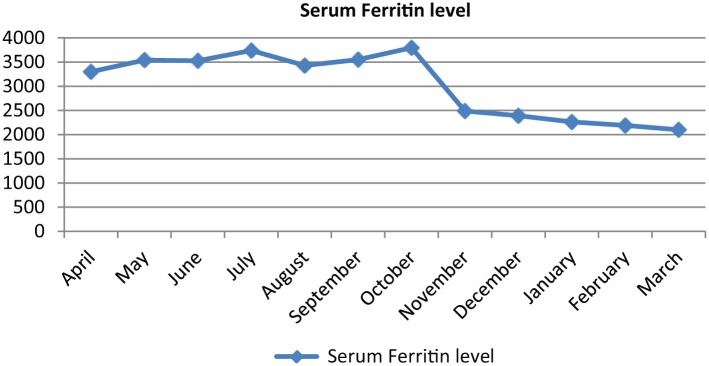
Serum ferritin level in ng/mL over the last year.

In October 2015, he received IV deferoxamine 5 days per week for 1 month, after which the serum ferritin level started to decline gradually (Figure 1). He is currently on oral deferasirox and IV deferoxamine monthly.

## Discussion

Iron overload from chronic transfusion therapy can be extremely toxic. Excess transfusional iron is deposited in the liver, heart, and other organs as free iron, which can cause organ dysfunction and damage over time.^2^

There are no mechanisms that can remove excess iron load from the body. Classically, a unit of transfused blood contains 200 to 250 mg of iron. Thus, patients who are receiving an average of 2 to 4 units of blood monthly will have an iron intake of 5000 to 10 000 mg of iron per year.^3^

Inefficient iron chelation leads to death that occurs from cardiac failure or arrhythmia.^1^ Liver disease and endocrine disorders develop in thalassemic patients during childhood, and death from iron-induced cardiomyopathy can occur later in adult life.^4^ In patients with sickle cell anemia, iron deposits can occur in the liver, heart, and pancreas classically in late childhood.^5^

Ideally, patients should be examined before initiation of iron chelation therapy, along with a thorough history and determining the body’s iron overload by specific investigations.^6^

Chelation therapy is necessary to prevent the consequences of iron overload. Three chelators, deferoxamine, deferiprone, and deferasirox, are presently available.^7^ These new chelation options increase the likelihood of achieving a normal pattern of complication-free survival and quality of life.^8^

Our patient was diagnosed with Diamond-Blackfan anemia shortly after birth. Having refused to proceed with bone marrow transplantation, we started him on blood transfusion when the requirements increased with time. Despite the regular iron chelation program, he developed insulin-dependent diabetes mellitus and has had frequent episodes of epistaxis, which were proven to be due to a platelet function defect.

On the other hand, acquired platelet function defects are classified broadly into defects that are intrinsic or extrinsic to the platelets. Acquired platelets defect are due to medications, medical conditions, underlying hematologic diseases, and are more frequent than inherited causes of platelets defects.^9^

The commonest medication that causes acquired platelet dysfunction is aspirin; other medications include nonsteroidal anti-inflammatory drugs, the thienopyridines clopidogrel and prasugrel, and the phosphodiesterase inhibitors dipyridamole and cilostazol.^10^

Medical conditions that are associated with disorders in platelet function have been reported. Myeloproliferative disorders are associated with hemostatic complications with hemorrhagic complications being more commonly seen in patients with essential thrombocythemia or polycythemia vera.^11,12^ In patients with myelodysplastic syndrome, bleeding can result from dysfunctional platelets,^13^ and in multiple myeloma and Waldenstrom macroglobulinemia, paraproteins might lead to multiple hemostatic defects including platelets dysfunction.^14^

Platelet dysfunction has also been reported in patients with uremia, liver dysfunction, cardiac bypass procedures, sepsis/infections, leukemia, and conditions including disseminated intravascular coagulation.

Diamond-Blackfan anemia is occasionally associated with hematologic defects other than a deficiency of red blood cell progenitors. Buchanan et al concluded in a study that thrombocytosis or thrombocytopenia often occurs in patients with Diamond-Blackfan anemia but platelet function is normal.^15^

A specific diagnosis often requires more sophisticated tests of platelet function that may be available only in specialized laboratories.

The bleeding time and platelet function analyzer are the first to be measured. However, clinicians used to rule out von Willebrand disease followed by platelet aggregation studies that should be performed. More specific tests, in particular, flow cytometry, is used to measure αIIbβ3 activation or α-granule secretion via P-selectin (CD62P) expression.^16,17^

The treatment of choice in bleeding due to acquired platelets defect is platelet transfusion. However, other therapeutic agents are available. Adjunctive therapies (such as antifibrinolytics, microfibular collagen, fibrin glue, etc) and DDAVP remain the mainstay of therapy available at this time. Recombinant factor VIIa is effectively indicated and has a wide spectrum of uses in hemostatic defects due to platelet function defects and coagulopathy.^18^

On review of the literature, little was found regarding the direct correlation between hemochromatosis and acquired platelet function defects.

Popov et al reported in one study about myelodysplastic syndrome patients that there is a certain association of iron overload and high levels of reactive oxygen species, where reactive oxygen species represent an important parameter involved in platelet receptor activation. Patients with hemochromatosis have low platelet aggregation induced by thrombin; however, little is known about the effects of iron overload on platelet activation in myelodysplastic syndrome patients.^19^

Lynch and Soslau noticed while performing platelet aggregation studies on hemochromatotic blood samples that the increased serum iron levels associated with this disease almost completely inhibited γ-thrombin-induced platelet aggregation, and the authors concluded that it is unknown as to which specific clinical implications iron inhibition of γ-thrombin may have, but it is very possible that other conditions caused by hemochromatosis could be exacerbated by the inability of platelets to aggregate normally.^20^

## Conclusion

We herein report the case of a patient with acquired platelet function defect associated with iron overload as a consequence of chronic blood transfusion. Therefore, we emphasize the necessity of further studies to confirm direct correlation between iron overload as a causative agent and platelets dysfunction. And we recommend screening for platelet function in patients receiving chronic blood transfusion aiming at possible prevention of any life-threatening bleeding.
